# Mind the Gap: Exploring Parental Intentions, Actual Engagement, and Associated Outcomes in Tailored Digital Parent Training

**DOI:** 10.3390/pediatric18030064

**Published:** 2026-05-01

**Authors:** Or Brandes, Chen R. Saar, Orly Sapir-Budnero, Amit Baumel

**Affiliations:** Department of Community Mental Health, University of Haifa, Haifa 3498838, Israel; orachm01@campus.haifa.ac.il (O.B.); csaar@campus.haifa.ac.il (C.R.S.); osapirbu@campus.haifa.ac.il (O.S.-B.)

**Keywords:** digital parent training, content tailoring, user engagement, disruptive behavior

## Abstract

**Background/Objectives:** Digital parent training (DPT) programs offer scalable solutions for childhood disruptive behaviors but face significant engagement challenges. Although content tailoring may enhance outcomes, its clinical impact remains under-examined. This study aimed to (a) describe the correspondence between program recommendations, parental choices and engagement, and (b) examine how initial decisions are associated with subsequent engagement and therapeutic outcomes. **Methods:** A secondary analysis of three randomized trials included 151 parents of children (ages 3–7) with disruptive behaviors. Participants were classified as ‘Recommendation-Adherent’ (*n* = 63) or ‘Beyond-Recommendation’ (*n* = 88) based on whether initial content selections matched or exceeded program recommendations. Clinical outcomes (child behavior, parenting styles) and objective usage metrics were assessed at baseline and post intervention. **Results:** Many parents chose to expand the intervention scope beyond clinical recommendations (e.g., 91.5% selected the non-recommended Emotion Regulation module). However, this proactive initial intention did not increase objective engagement; groups did not differ significantly in total usage time, login days, or module completion rates. Although both groups showed comparable improvements in child behavior, intending to adhere to the recommended pathway was associated with significantly greater reductions in permissive parenting (laxness; *p* = 0.029) after adjusting for baseline differences. **Conclusions:** The findings highlight a discrepancy between parents’ intent to expand intervention scope and their actual engagement capacity. While the decision to adhere to a tailored pathway was associated with specific improvements in permissive parenting, the observational nature of the study precludes causal claims. Nevertheless, the results suggest that guided tailoring may serve as a protective function against choice overload. Aligning program demands with the practical realities of parental effort could help families focus finite energy on essential clinical targets.

## 1. Introduction

Digital parent training programs (DPTs) have emerged as a scalable and cost-effective approach for treating childhood disruptive behaviors, providing parents with remote access to evidence-based practices [1,2,3,4]. By removing structural barriers, DPTs expand clinical reach to populations that vary in baseline motivation and clinical needs [5,6,7]. However, this enhanced accessibility is frequently undermined by significant engagement challenges. Self-guided digital mental health interventions (DMHIs) face attrition rates exceeding 50% [8,9], which compromises therapeutic impact and highlights the need for strategies to sustain user adherence and maximize clinical outcomes [8,9,10,11,12].

Efforts to overcome these engagement challenges are largely informed by two complementary frameworks: Self-Determination Theory (SDT) and Cognitive Load Theory. SDT [13] posits that human motivation and well-being are sustained through the satisfaction of three basic psychological needs: autonomy, competence, and relatedness. In health behavior contexts, autonomy support, which involves providing individuals with meaningful choices and acknowledging their perspective, has been consistently associated with greater engagement, sustained motivation, and improved outcomes [14,15]. Applied to DMHIs, this perspective suggests that empowering users to shape their intervention experience may facilitate engagement and adherence [16,17,18]. However, SDT also emphasizes that autonomy does not operate in isolation. It must be supported by a structure that fosters competence [19]. Autonomy without adequate scaffolding can undermine rather than enhance self-regulation, particularly when individuals lack the expertise to make informed choices about their treatment [20,21].

The potential risks of providing autonomy without such structure are best understood through Cognitive Load Theory, which provides a complementary lens for examining how choice architecture affects engagement in DMHIs. Research on choice overload has demonstrated that expanding the number of options available to decision-makers can paradoxically reduce motivation, impair decision quality, and diminish satisfaction [22,23,24]. In the context of digital health, parents navigating a modular intervention face decisions about which content to select, drawing on the same cognitive resources needed for the therapeutic process itself [25]. When the number of modules exceeds what is clinically necessary, parents may experience increased cognitive burden, diffused attention across content areas, and reduced capacity to deeply engage with the most relevant material for their specific needs. This perspective suggests that filtering intervention content to match an individual’s clinical needs may protect finite parental cognitive and motivational resources.

Content tailoring in DMHIs represents a design approach that holds the capacity to balance these two theoretical concerns. When applied effectively, it can preserve user agency while managing cognitive demands through personalized content selection [26,27]. Systematic tailoring can adapt intervention components (e.g., content, sequencing, dosage) to the specific characteristics and clinical needs of each user, with the aim of increasing perceived relevance while reducing exposure to non-essential content [16,27]. Tailoring strategies, including rule-based decision systems and user-driven selection, are increasingly common in DMHIs, though empirical evidence for their effectiveness remains mixed [28,29]. Some studies show that adaptive pathways enhance engagement and outcomes beyond traditional fixed protocols [17,18], while others offer only preliminary support [26,27].

The relationship between engagement, adherence, and outcomes in DMHIs further underscores the importance of how users interact with intervention content. Systematic reviews indicate that greater adherence to prescribed treatment protocols is associated with better outcomes across a range of DMHIs [30,31]. Furthermore, design features promoting focused engagement, including personalization, reminders, and structured feedback, predict higher adherence rates [32,33]. These findings suggest that the pattern of engagement, not merely its volume, may be critical for clinical benefit.

Despite the theoretical promise of combining structured tailoring with user autonomy, empirical evaluations of this balance within DPTs remain notably limited. Existing research in the field of DPTs primarily reflects fixed and self-paced structures, characterized by adaptations such as coach-guided feedback or flexible module sequencing. Specifically, there is a lack of evidence regarding the effectiveness of tailoring intervention content based on parents’ initial reports of their own parenting difficulties. Moreover, it remains unclear whether a parent’s decision to follow or diverge from a clinically informed recommendation affects therapeutic outcomes, and if so, whether this effect is consistent with the predictions of SDT and Cognitive Load Theory perspectives.

The present study addresses this gap by evaluating the clinical implications of content tailoring based on parents’ self-reported assessment of family needs at the start of the intervention. In this framework, the program generates a suggested path of individualized module recommendations based on responses to a short intake questionnaire. Parents, however, retain the autonomy to either adhere to this suggested path or to expand their intervention scope by selecting additional modules. This design creates a naturalistic comparison between parents who followed the clinically informed recommendations and those who exercised their autonomy to go beyond them, offering an empirical test of the tension between guided structure and user-driven choice.

By analyzing these engagement patterns, we sought to address two questions:

1. The engagement pipeline from recommendation to completion: We examined the correspondence between the program’s suggested path, pre-program parental module selection, and actual module completion to understand how parents navigated the tension between tailored and self-directed content choices.
2. The clinical value of adhering to tailored recommendations: We examined whether parents who followed the program’s suggested path, which was based on their reported parenting challenges and child symptoms, achieved greater therapeutic gains compared to those who chose to expand the intervention scope.

## 2. Materials and Methods

### 2.1. Research Design

The present study is a secondary analysis involving participants from three distinct randomized controlled trials (RCTs) conducted between 2023 and 2025. Specifically, the sample consists of participants from three trials [33,34,35], evaluating the efficacy of DPT for children with disruptive behaviors, specifically focusing on the integration of ‘therapeutic persuasiveness’ (TP) features within the DPT. The current sub-study focused specifically on a cohort of participants who received the enhanced DPT (incorporating TP features) in either guided or unguided formats. Outcome measures were assessed using a repeated-measures design at baseline (T1) and post intervention (T2). The studies were approved by the University of Haifa Institutional Review Board (Approval No. 058/22, 342/22, 418/23).

### 2.2. Participants and Recruitment

Participants were recruited via targeted social media advertising. Inclusion criteria were as follows: (1) being a parent of a child aged 3–7 years; (2) child exhibition of elevated disruptive behavior, defined by an Eyberg Child Behavior Inventory (ECBI) Intensity score ≥ 132 or Problem score ≥ 15; and (3) having a smartphone. Exclusion criteria included (1) concurrent professional treatment for the child’s behavioral/emotional issues or parental participation in other training programs; and (2) a known diagnosis of intellectual disability or developmental delay in the child. Eligible parents underwent a telephone screening interview to confirm eligibility. Subsequently, participants provided electronic informed consent and completed a baseline assessment battery.

**Figure A1:**
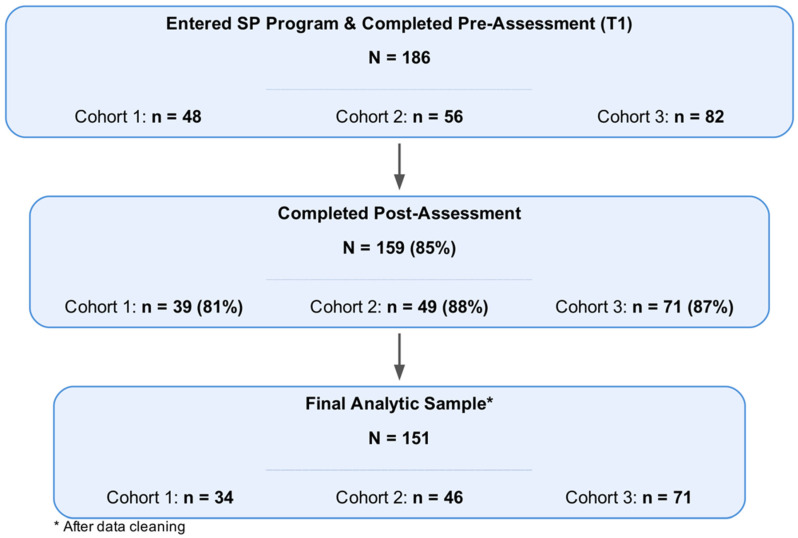
Flow of participants through study stages across three cohorts.

A total of 186 eligible parents entered the program. Of these, 159 completed the post-intervention assessment, reflecting an 85% retention rate. Following data cleaning, the final analytic sample consisted of 151 participants (see Figure A1 for participant flow). The present study used a strict complete-case analysis approach, restricting the statistical evaluation exclusively to participants who provided data at both time points. No missing data imputation was performed for those who dropped out prior to completion.

### 2.3. Overview of Intervention

The intervention was grounded in the theoretical framework asserting that parental interaction patterns serve as primary catalysts for child behavior change [33]. To ensure that the intervention was precisely tailored to the specific clinical needs and parenting skills of each participant, the program used a rule-based content tailoring process, where program content and sequence were customized based on a short intake questionnaire.

**Figure A2:**
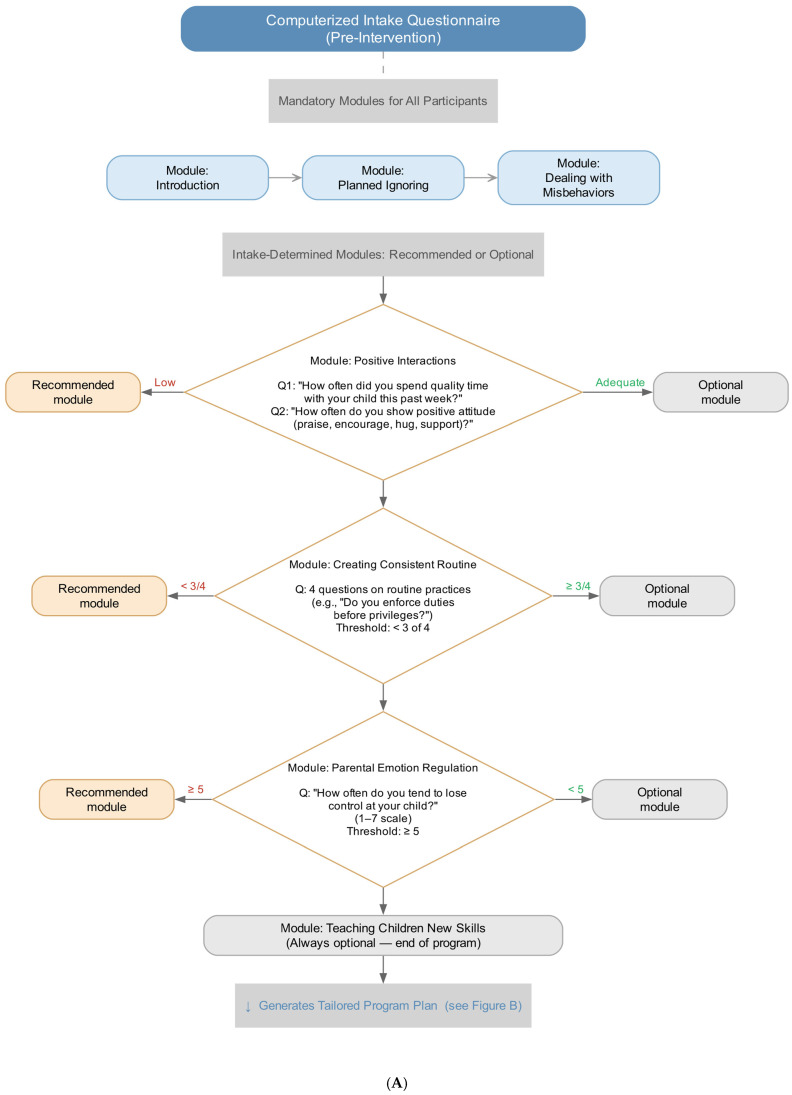

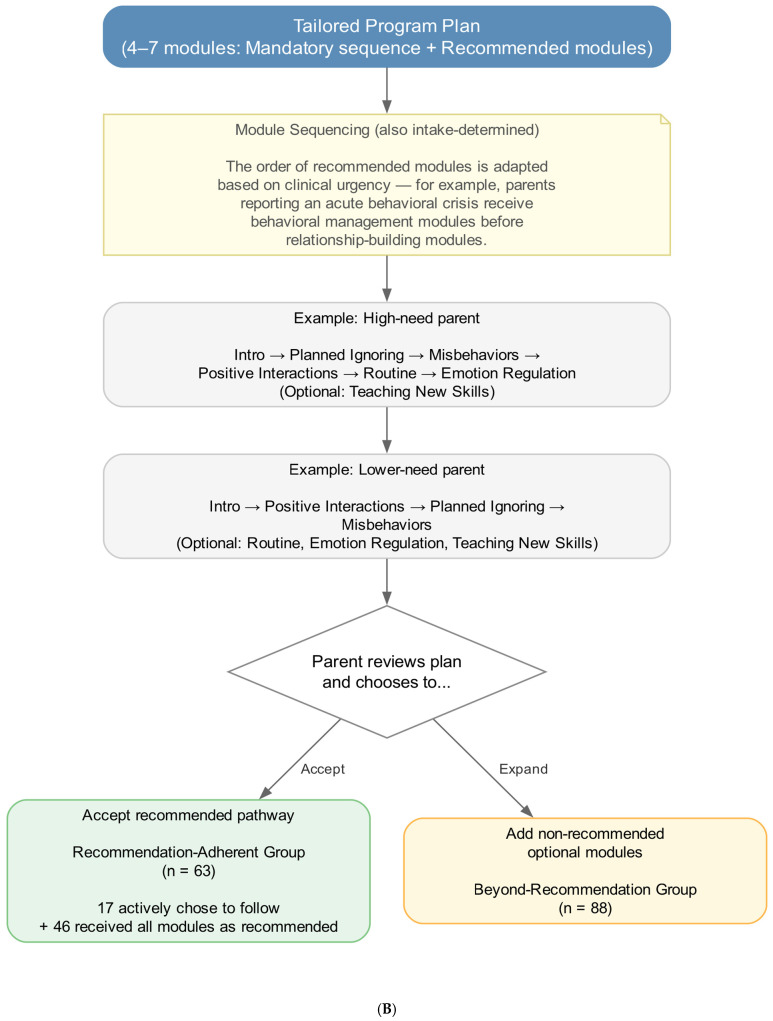
The Intervention Tailoring Process. (**A**) A logic model for intake-determined module recommendations; (**B**) module sequencing examples and final group classification based on participant choices.

Based on the assessment, the program presents each participant with a recommended program plan (see Figure A2 for the tailoring logic and group assignment). Of the seven available modules, three were mandatory for all participants (Introduction, Planned Ignoring, Dealing with Misbehaviors), and three additional modules were conditionally recommended based on specific score thresholds from the intake assessment (Positive Interactions, Routine, Parental Emotion Regulation). The seventh module, Teaching Children New Skills, was always offered as an optional module at the end of the program and was never explicitly recommended by the program. The sequence of the assigned modules was also adapted based on clinical urgency. For instance, parents reporting an acute behavioral crisis received behavioral management modules before relationship-building modules. Despite these tailored recommendations, participants retained full autonomy to adjust their final selection before beginning the intervention. This enabled them to either engage with additional non-recommended optional modules or focus exclusively on the program’s suggested path.

Additionally, the program incorporated ‘therapeutic persuasiveness’ (TP) features [33]. Each module consisted of a 10 to 25 min learning phase, followed by a 1- to 2-week practice phase. These design elements, including ‘call to action’ triggers, automated feedback, and digital monitoring tools, were specifically intended to translate therapeutic concepts into daily parenting practice.

### 2.4. Measures

Data were collected through a combination of self-reported questionnaires and automated program usage metrics. Demographic information was gathered during the baseline assessment. The self-reported measures were delivered via the ‘Qualtrics’ platform (Qualtrics, Provo, UT, USA). Child behavior and parenting-related variables were provided by the ‘lead parent’, that is, the primary caregiver responsible for engaging with the intervention.

The Eyberg Child Behavior Inventory (ECBI) [36] was used to evaluate disruptive child behaviors, with the Intensity Scale (ECBI-I) serving as the primary outcome measure. This 36-item instrument assesses the frequency of common behavioral problems as reported by parents. Each item is rated on a 7-point Likert scale, ranging from 1 (never) to 7 (always) [37]. The ECBI-I is a well-validated tool, recognized for its capacity to differentiate between clinical and non-clinical behavior levels and its sensitivity to symptomatic changes following clinical interventions [37,38]. In the current study, the scale demonstrated good internal consistency, with a Cronbach’s alpha of 0.80.

Parenting Scale (PS) [39] was employed to assess parental disciplinary styles through a series of hypothetical scenarios involving child misbehavior. For each item, parents indicate their typical reaction on a 7-point Likert scale, where higher scores represent more effective parenting and lower scores represent more dysfunctional responses (reverse scored). This study used two specific subscales: Over-reactivity (PS-OV), comprising 11 items that measure harsh or emotionally driven reactions, and Laxness (PS-LA), consisting of 10 items that evaluate inconsistent or permissive discipline. In this sample, both subscales demonstrated adequate internal consistency, with Cronbach’s alpha coefficients of 0.75 for Over-reactivity and 0.83 for Laxness.

Parenting Tasks Checklist (PTC) [40] was used to measure task-specific parental self-efficacy. Specifically, the Behavioral Self-Efficacy subscale was used, consisting of 6 statements that evaluate a parent’s confidence in managing specific child behaviors. Respondents rate their level of certainty for each task on a scale from 0 (“Certain I can’t do it”) to 100 (“Certain I can do it”). In the current study, this subscale exhibited high internal consistency, with a Cronbach’s alpha of 0.91.

### 2.5. Data Analysis

Participants were classified into two groups based on their engagement with the program’s module recommendations. The ‘Recommendation-Adherent’ group comprised parents who opted to strictly follow the recommended clinical pathway. In contrast, the ‘Beyond-Recommendation’ group included participants who, during the initial design phase, declared their intent to expand their intervention scope by selecting additional modules beyond those recommended by the system.

The ‘Recommendation-Adherent’ group was composed of two subgroups: (a) parents whose decisions precisely matched the partial recommendations provided, and (b) parents who followed the recommendations but were assigned all modules, thereby precluding the possibility of exceeding them. To ensure the homogeneity of this combined group, sensitivity analyses were conducted prior to merging to verify that these participants shared similar baseline levels and clinical trajectories (see ‘sensitivity analysis’ in the Section 3).

Statistical analysis was conducted using SPSS (version 27; IBM Corp; Armonk, NY, USA) in several stages. First, to evaluate the initial comparability of the groups and identify potential baseline imbalances, independent-samples t-tests and Chi-square tests (or Fisher’s exact tests) were used to compare socio-demographic characteristics and baseline clinical scores (Table 1). Second, independent-samples t-tests were performed to compare the groups on usage time, login frequency, and module completion rates. Effect sizes were estimated using Cohen’s *d*.

Third, to evaluate the clinical value of adhering to tailored recommendations, a series of repeated-measures General Linear Models (GLMs) were conducted. A complete-case GLM approach was selected over mixed-effects modeling because the data contained exactly two measurement occasions (pre and post) with no partial missingness across waves for the analyzed sample. Furthermore, participants were pooled across trials, with trial membership included as a covariate rather than a hierarchical nesting factor. For each outcome (child behavior, parenting styles, and self-efficacy), Time (baseline vs. post-intervention) was entered as a within-subjects factor, and Group (Adherent vs. Beyond) as a between-subjects factor. To ensure robust adjustment and account for baseline differences, all models included five covariates: child gender, trial membership, delivery format (guided vs. unguided), baseline over-reactivity, and baseline laxness. As a sensitivity check, preliminary analyses confirmed that neither trial membership nor delivery format significantly predicted any outcome, further supporting the pooled analytic approach. Significant interactions were decomposed using simple-effects analyses to determine the source of the divergent trajectories.

## 3. Results

Background characteristics are presented in Table 1. The study included 151 families with children with behavior problems (61.6% boys, mean age = 5.08 years). Preliminary analyses indicated no differences between the two groups except for child gender and parenting overreactive styles. Specifically, the ‘Recommendation-Adherent’ group featured a higher proportion of girls compared to the ‘Beyond-Recommendation’ group. Additionally, parents from the ‘Beyond-Recommendation’ group reported significantly lower levels of over-reactivity and marginally lower levels of laxness at baseline compared to the ‘Recommendation-Adherent’ group.

Table 2 presents the module engagement pipeline for the three conditional modules, tracking the correspondence between program recommendations, parental pre-program selections, and actual module completion. For all three modules, the rate at which parents decided to include them prior to starting the program exceeded the rate at which those modules were recommended by the algorithm. This gap was most pronounced for the Emotion Regulation module, where 97% of participants across both groups elected to include it, compared to 61% who received a formal recommendation. The pattern was consistent for Effective Routines (83% decided vs. 62% recommended) and Positive Interactions (83% decided vs. 54% recommended), indicating a broad parental preference for a comprehensive intervention scope regardless of the algorithm’s clinical assessment.

Completion data revealed that the majority of parents who selected modules followed through on their choices, though completion rates fell below selection rates across all modules and both groups. Completion was highest for the Emotion Regulation module (85% overall) and lowest for Effective Routines (62%). These patterns were broadly similar between the Adherent and Beyond-Recommendation groups. Consistent with the convergence in module selections, no significant differences emerged between the two groups on any objective engagement metric, including total use time, login days, and module completion rates (all *p*s > 0.11; see Table 3).

Table 4 presents the pre-to-post intervention changes in parenting styles (over-reactivity and laxness), child disruptive behaviors (ECBI Intensity), and parental self-efficacy (PTC-Behavior). To account for identified baseline differences between the groups, all repeated-measures GLMs included five covariates: child gender, trial membership, delivery format (guided vs. unguided), baseline over-reactivity, and baseline laxness.

**Figure 1:**
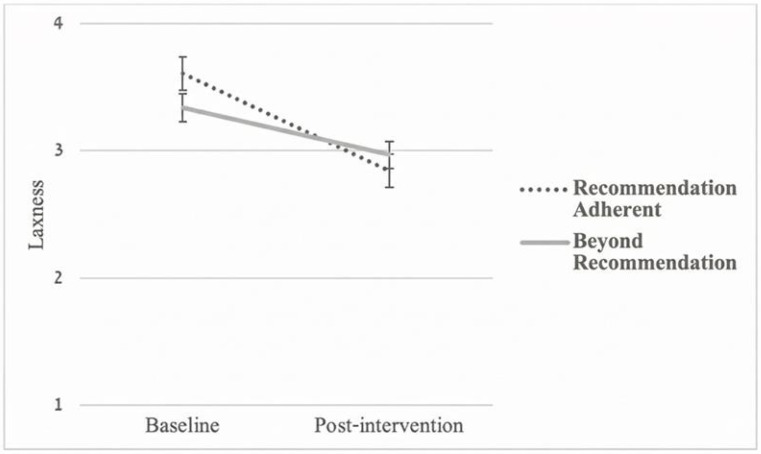
Changes in Laxness From Baseline to Post-Intervention by Recommendation Groups.

**Figure 2:**
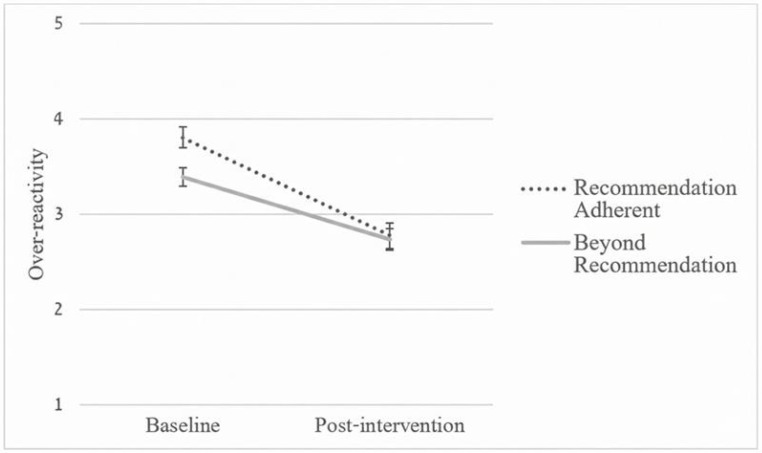
Changes in Over-Reactivity from Baseline to Post-Intervention by Recommendation Groups.

Regarding parenting styles, a significant Time × Group interaction emerged for laxness (*F*(1, 144) = 4.87, *p* = 0.029, *η_p_*^2^ = 0.033), indicating that the ‘Recommendation-Adherent’ group exhibited a significantly steeper decline in laxness compared to the ‘Beyond-Recommendation’ group (see Figure 1). The Time × Group interaction for over-reactivity, which was significant in unadjusted models (*p* = 0.025), was attenuated and no longer statistically significant after adjusting for baseline parenting styles (*F*(1, 144) = 1.23, *p* = 0.270, *η_p_*^2^ = 0.008; see Figure 2). This attenuation is consistent with regression to the mean, as the Adherent group reported significantly higher baseline over-reactivity (*M* = 3.87 vs. 3.35, *p* < 0.001) and thus had more room for measured improvement.

For child behavior, a significant main effect of time emerged for ECBI Intensity (*F*(1, 144) = 5.45, *p* = 0.021), reflecting substantial reductions in disruptive behaviors across the entire sample. However, the Time × Group interaction was non-significant (*p* = 0.758), indicating that the magnitude of clinical improvement in child behavior was comparable between the two groups. Analyses of parental self-efficacy (PTC-Behavior) yielded no significant Time × Group interaction (*p* = 0.803).

### Sensitivity Analyses

To ensure the homogeneity of the ‘Recommendation-Adherent’ group (*n* = 63), sensitivity analyses were conducted between the two constituent subgroups: (a) parents whose selections precisely matched the partial recommendations provided (“Not all modules recommended”) (*n* = 17), and (b) parents who were assigned to a full curriculum, thereby precluding the possibility of exceeding the recommendations (“All modules recommended”) (*n* = 46).

Analyses of usage metrics revealed that parents in the ‘All modules recommended’ subgroup exhibited higher number of unique logins (*M* = 31.74, *SD* = 14.73) compared to the ‘Not all modules recommended’ subgroup (*M* = 25.29, *SD* = 6.48), *t* (59.0) = 2.40, *p* = 0.019. Similarly, the ‘All modules recommended’ subgroup recorded more login days (*M* = 27.96, *SD* = 12.71 vs. *M* = 23.18, *SD* = 5.55), *t* (59.1) = 2.07, *p* = 0.043, and higher module completion rates (*M* = 0.99, *SD* = 0.04 vs. *M* = 0.85, *SD* = 0.24), *t* (51.3) = 3.79, *p* < 0.001. These differences likely reflect the higher clinical need and greater program dosage assigned to the “All modules recommended” subgroup. No significant differences were found for total usage time or completed sessions, with t-values ranging from 0.66 to 1.80 (*p* = 0.077 to 0.355). No significant differences emerged between the two subgroups regarding baseline levels or pre-to-post changes across any parenting or child behavior outcomes (all *p* ≥ 0.170). These results support the consolidation of the two subgroups into a single ‘Recommendation-Adherent’ category for the primary analyses.

## 4. Discussion

The present study sought to examine the interplay between content tailoring based on parents’ initial reports of their own parenting difficulties and parental autonomy within DPTs. Specifically, we examined how the correspondence between program recommendations and parental pre-intervention decisions was associated with engagement and therapeutic outcomes. Our findings revealed a gap between parents’ initial choice to expand the intervention scope and their actual engagement patterns; specifically, the intention to complete more modules did not translate into increased objective usage. Furthermore, the initial intention to follow the program’s suggested path was associated with greater improvements in permissive parenting style (laxness), compared to the initial intention to expand the intervention scope. These results align with emerging perspectives suggesting that user autonomy may not function as an unqualified benefit in digital health. Rather, it is plausible that content tailoring defined by clinical considerations serves a protective function, aligning intervention demands with the user’s finite capacity to invest effort [41,42].

A key finding of this study was that a high percentage of parents chose to expand the intervention content beyond the program recommendations when provided the opportunity to do so during the initial phase. This trend was most pronounced for the Emotion Regulation module, which was selected by 91.5% of parents for whom it was not clinically recommended. However, our findings revealed a notable gap between this stated willingness and actual engagement patterns. In practice, despite their proactive intentions, parents generally completed fewer modules than they had initially planned. Specifically, no significant differences were found in total usage time, login days, or completion rates between parents who initially intended to adhere to the program recommendations and those who intended to expand them.

Although both groups demonstrated comparable improvements in child behavior, the initial intention to adhere to the recommended pathway was associated with more favorable changes in specific parenting domains. In unadjusted models, the Adherent group exhibited what appeared to be sharper declines in both over-reactivity (*p* = 0.025) and laxness (*p* = 0.004). However, after adjusting for baseline differences, only the greater improvement in laxness remained statistically significant (*p* = 0.029). Because the Adherent group reported higher over-reactivity at baseline, their seemingly steeper decline in the unadjusted model is consistent with regression to the mean rather than a true effect of the tailored pathway.

The observed association between guided tailoring and laxness improvements is consistent with the findings of Savir and Baumel (2025) [43], who demonstrated that individuals’ estimations of their capacity to invest effort significantly influence their interaction with mental health interventions. It is plausible that the act of initiating an intervention is accompanied by a degree of initial optimism, leading some parents to make proactive choices that may exceed their actual capacity for sustained, longitudinal engagement. Viewed through the lens of Cognitive Load Theory, expanding the program’s scope may have resulted in a misallocation of finite cognitive resources. Specifically, by adding non-essential modules to a limited effort budget, parents in the Beyond-Recommendation group might have diluted their focus, diverting attention toward personally chosen content rather than concentrating on the core behavioral strategies most critical to their clinical needs. In contrast, the tailored structure might have acted as a necessary scaffolding for the Adherent group, consistent with Self-Determination Theory’s assertion that autonomy requires supportive structure to be effective [19].

### 4.1. Clinical Implications

These findings suggest that DPT design should account for the discrepancy between parents’ initial high motivation and their actual capacity for sustained engagement. While parents often enter interventions intending to maximize their learning through additional content, these intentions may not anticipate the emergence of competing life demands and inevitable fatigue. To bridge this gap, platforms should prioritize managing expectations regarding time and effort from the outset. By encouraging realistic commitments and emphasizing core, clinically indicated tasks over a broader curriculum, developers can help parents protect their finite energy and focus it where it is most likely to yield behavioral change.

### 4.2. Limitations

Several limitations warrant consideration. First, because this study used an observational design in a secondary analysis, participants were not randomized to follow or exceed the program recommendations. Group membership was defined by parental choice, rendering the findings susceptible to self-selection bias. Although baseline differences in parenting styles and child gender were statistically controlled, unmeasured confounders such as initial motivation, parental anxiety, or perfectionism may have influenced both module selection and clinical trajectories. Therefore, these findings demonstrate associations rather than causal effects.

Second, the construction of the ‘Recommendation-Adherent’ group involved a necessary methodological trade-off. Within this group, only 17 parents actively chose to adhere to a partial recommendation, meaning that they were recommended fewer than three modules and chose not to add any. Due to the statistical limitations of this small subsample, these participants were pooled with parents who were recommended all three optional modules and thus had no structural opportunity to exceed the recommendation. While sensitivity analyses demonstrated no significant differences between these two subgroups, justifying their consolidation for the current analysis, they remain conceptually distinct. One reflects adherence by choice while the other reflects a structural constraint. Future research with larger samples should investigate these subgroups separately to further untangle the roles of parental agency versus program structure.

Third, the present study relied exclusively on participants who completed assessments at both time points. This precluded an evaluation of attrition data and limited our ability to determine whether dropout rates differed between the subgroups. Furthermore, the study could have benefited from the analysis and reporting of additional, more granular usage data (such as time spent on each module). Such metrics could have provided a deeper and broader understanding of the distribution of effort between recommended and non-recommended content.

## 5. Conclusions

This study contributes to the literature on DPTs by highlighting the complex relationship between parental choice and structured clinical tailoring. The findings illustrate a clear discrepancy between parents’ initial intent to expand the intervention scope and their actual engagement capacity. While the results suggest that the decision to follow a tailored pathway is associated with specific clinical benefits, particularly in reducing permissive parenting, the observational nature of this study precludes definitive causal claims. Ultimately, these results underscore the importance of designing digital interventions that respect user agency while protecting parents from the potential burden of choice overload. Future research utilizing randomized adaptive designs is necessary to establish whether such structured tailoring can systematically optimize clinical gains across diverse populations. By better aligning program demands with the practical realities of parental effort, developers can better support families through sustainable digital interventions.

## Figures and Tables

**Table 1:** Differences in Baseline Characteristics Between Recommendation-Adherent and Beyond-Recommendation Groups.

Variable	Recommendation-Adherent (*n* = 63)	Beyond-Recommendation (*n* = 88)	Total (*N* = 151)	Statistic	*p*
Continuous variables, *M* (*SD*)					
Parent age ^1^	36.06 (3.41)	36.96 (4.23)	36.58 (3.92)	*t* = 1.40	0.163
Child age	5.13 (1.12)	5.05 (1.29)	5.08 (1.22)	*t* = 0.37	0.714
Number of children in family	2.52 (1.01)	2.60 (0.88)	2.57 (0.94)	*t* = 0.51	0.613
Baseline Variables					
ECBI Intensity	162.97 (22.42)	165.19 (20.13)	164.26 (21.07)	*t* = 0.64	0.524
PTC—Behavior	48.26 (24.14)	53.75 (19.76)	51.46 (21.78)	*t* = 1.53	0.128
Parenting Style—Over-reactivity	3.87 (0.83)	3.35 (0.91)	3.56 (0.91)	*t* = 3.62	<0.001
Parenting Style—Laxness	3.64 (1.00)	3.32 (1.03)	3.45 (1.03)	*t* = 1.91	0.058
Categorical variables, *n* (%)					
Leading parent gender				Fisher	1.000
Male	3 (4.8)	4 (4.5)	7 (4.6)		
Female	60 (95.2)	84 (95.5)	144 (95.4)		
Child gender				*χ*^2^ = 13.43	<0.001
Male	28 (44.4)	65 (73.9)	93 (61.6)		
Female	35 (55.6)	23 (26.1)	58 (38.4)		
Participating parents				*χ*^2^ = 2.17	0.141
Both parents	37 (58.7)	41 (46.6)	78 (51.7)		
One parent	26 (41.3)	47 (53.4)	73 (48.3)		
Education ^1^				*χ*^2^ = 0.73	0.394
High school	11 (17.5)	11 (12.5)	22 (14.6)		
Above high school	52 (82.5)	77 (87.5)	129 (85.4)		
Household income ^2^				*χ*^2^ = 2.62	0.269
<15 K	15 (23.8)	12 (13.6)	27 (17.9)		
15–18 K	15 (23.8)	25 (28.4)	40 (26.5)		
>18 K	33 (52.4)	51 (58.0)	84 (55.6)		
Religiosity				*χ*^2^ = 1.92	0.382
Secular	36 (57.1)	44 (50.0)	80 (53.0)		
Traditional	20 (31.7)	27 (30.7)	47 (31.1)		
Religious	7 (11.1)	17 (19.3)	24 (15.9)		
Hours of work/study per week ^1^				*χ*^2^ = 0.11	0.947
<10 h	9 (14.3)	11 (12.5)	20 (13.2)		
10–29 h	8 (12.7)	11 (12.5)	19 (12.6)		
>30 h	46 (73.0)	66 (75.0)	112 (74.2)		

Note. ^1^ Refers to the leading parent of the intervention. ^2^ In Israeli Shekels (ILS).

**Table 2:** Module Engagement Pipeline: Recommended, Decided, and Completed by Group.

Optional Module	Stage ^1^	Adherent (*n* = 63)	Beyond (*n* = 88)	Total (*N* = 151)
Effective Routines	Recommendation	52 (83%)	41 (47%)	93 (62%)
	Decision	52 (83%)	74 (84%)	126 (83%)
	Completion	37 (59%)	57 (65%)	94 (62%)
Emotion Regulation	Recommendation	61 (97%)	31 (35%)	92 (61%)
	Decision	61 (97%)	85 (97%)	146 (97%)
	Completion	58 (92%)	70 (80%)	128 (85%)
Positive Interaction	Recommendation	52 (83%)	29 (33%)	81 (54%)
	Decision	52 (83%)	74 (84%)	126 (83%)
	Completion	40 (63%)	62 (70%)	102 (68%)

^1^ Recommendation = program output; Decision = participant’s pre-program module selection; Completion = module finished based on platform usage logs.

**Table 3:** Differences between groups in program usage metrics.

	Recommendation Adherent (*n* = 63)	Beyond Recommendation (*n* = 88)			
	*M (SD)*	*M (SD)*	*t* (149)	*p*	*d*
Use time (minutes)	140.41 (59.46)	132.26 (69.23)	0.76	0.451	0.13
Login days	26.67 (11.39)	23.78 (10.79)	1.58	0.116	0.26
Unique Logins	30.00 (13.29)	27.34 (13.04)	1.23	0.222	0.20
Sessions completed	5.79 (1.49)	5.74 (1.65)	0.21	0.834	0.04
Module Completion Rate	0.888 (0.216)	0.859 (0.233)	0.77	0.442	0.13

**Table 4:** Descriptive statistics and differences from baseline to post intervention between the recommendation groups.

	Recommendation-Adherent		Beyond-Recommendation						
	(*n* = 63)		(*n* = 88)		ANOVA Effects			Effect Size	
	Baseline *M* (*SD*)	Post *M* (*SD*)	Baseline *M* (*SD*)	Post *M* (*SD*)	Time Effect	Group Effect	Time × Group Effect	B [95% CI]	Cohen’s *d*
Parenting Style									
Over-reactivity	3.87 (0.83)	2.82 (1.19)	3.35 (0.91)	2.71 (0.91)	*F* (1, 144) = 0.12, *p* = 0.731, *η_p_*^2^ = 0.001	*F* (1, 144) = 1.23, *p* = 0.270, *η_p_*^2^ = 0.008	*F* (1, 144) = 1.23, *p* = 0.270, *η_p_*^2^ = 0.008	0.19 [−0.15, 0.54]	0.22
Laxness	3.64 (1.00)	2.85 (1.01)	3.32 (1.03)	2.96 (1.00)	*F* (1, 144) = 0.03, *p* = 0.867, *η_p_*^2^ = 0.001	*F* (1, 144) = 4.87, *p* = 0.029, *η_p_*^2^ = 0.033	*F* (1, 144) = 4.87, *p* = 0.029, *η_p_*^2^ = 0.033	0.31 [0.03, 0.59]	0.43
ECBI Intensity	162.97 (22.42)	134.11 (34.03)	165.19 (20.13)	139.23 (27.43)	*F* (1, 144) = 5.45, *p* = 0.021, *η_p_*^2^ = 0.036	*F* (1, 144) = 0.27, *p* = 0.604, *η_p_*^2^ = 0.002	*F* (1, 144) = 0.10, *p* = 0.758, *η_p_*^2^ = 0.001	1.70 [−9.17, 12.57]	0.06
PTC—Behavior	47.80 (24.05)	66.85 (22.91)	53.75 (19.76)	70.07 (20.80)	*F* (1, 143) = 0.88, *p* = 0.351, *η_p_*^2^ = 0.006	*F* (1, 143) = 1.18, *p* = 0.278, *η_p_*^2^ = 0.008	*F* (1, 143) = 0.06, *p* = 0.803, *η_p_*^2^ = 0.001	−1.18 [−10.53, 8.17]	0.05

Note. Values are from repeated-measures GLMs with Time × Group interactions, controlling for child gender, trial, delivery format, and baseline over-reactivity and laxness. B = unstandardized interaction coefficient (positive = greater Adherent improvement); Cohen’s *d* = B/residual SD. Group and Time × Group effects are equivalent because baselines were covaried. PTC analyses: *n* = 150 (one missing post-value). Significant effects (*p* < 0.05) in boldface.

## Data Availability

The data are not publicly available due to privacy reasons. All the anonymized data can be made available at the specific request to the corresponding author at e-mail abaumel@univ.haifa.ac.il.

## Abbreviations

The following abbreviations are used in this manuscript:

DPTs	Digital Parent training programs
SDT	Self-Determination Theory
PS	Parenting Scale
PTC	Parenting Task Checklist
ECBI	Eyberg Child Behavior Inventory
DMHIs	digital mental health interventions
RCTs	randomized controlled trials
TP	therapeutic persuasiveness
GLMs	General Linear Models
